# Neutrophil heterogeneity and its role in infectious complications after severe trauma

**DOI:** 10.1186/s13017-019-0244-3

**Published:** 2019-05-29

**Authors:** Lillian Hesselink, Roy Spijkerman, Karlijn J. P. van Wessem, Leo Koenderman, Luke P. H. Leenen, Markus Huber-Lang, Falco Hietbrink

**Affiliations:** 1 Department of Trauma Surgery, University Medical Centre Utrecht, Utrecht, The Netherlands; 2 Laboratory of Translational Immunology and Department of Respiratory Medicine, University Medical Centre Utrecht, Utrecht, The Netherlands; 3 Institute of Clinical and Experimental Trauma Immunology, University Hospital of Ulm, Ulm, Germany

**Keywords:** Neutrophil, Trauma, Infection, Immune response

## Abstract

**Background:**

Trauma leads to a complex inflammatory cascade that induces both immune activation and a refractory immune state in parallel. Although both components are deemed necessary for recovery, the balance is tight and easily lost. Losing the balance can lead to life-threatening infectious complications as well as long-term immunosuppression with recurrent infections. Neutrophils are known to play a key role in these processes. Therefore, this review focuses on neutrophil characteristics and function after trauma and how these features can be used to identify trauma patients at risk for infectious complications.

**Results:**

Distinct neutrophil subtypes exist that play their own role in the recovery and/or development of infectious complications after trauma. Furthermore, the refractory immune state is related to the risk of infectious complications. These findings change the initial concepts of the immune response after trauma and give rise to new biomarkers for monitoring and predicting inflammatory complications in severely injured patients.

**Conclusion:**

For early recognition of patients at risk, the immune system should be monitored. Several neutrophil biomarkers show promising results and analysis of these markers has become accessible to such extent that they can be used for point-of-care decision making after trauma.

## Background

Both mortality and morbidity after trauma have globally decreased in the past decades [1]. However, trauma remains the leading cause of death in people under the age of 40 worldwide [2, 3]. The reason that more patients survive the initial trauma nowadays is mainly due to advances in (surgical) hemorrhage control and resuscitation [4]. However, after this first critical phase, patients can deteriorate again due to immune-related complications, such as an overwhelming immune response, severe infections, or recurrent infections later on [5, 6].

After trauma, the injury induces an immediate innate immune response to protect disrupted barriers from pathogens, to clear tissue damage, and to induce healing [7]. Many humoral and cellular mediators including leukocytes, the coagulation, and complement cascades strictly regulate these processes. However, sometimes these processes become dysregulated and severe immune mediated complications can occur [8]. Neutrophils are the most abundant leukocytes of all innate effector cells, and the first responders to tissue damage and invading pathogens [9]. These cells are able to internalize and kill microbes, as well as to perform tissue debridement and attract monocytes to initiate healing [10]. Therefore, neutrophils are known to play a key role in the post-traumatic immune response [8, 11]. Moreover, neutrophils do not only sense and clear molecular danger but also respond to many soluble factors and it is likely that neutrophils therefore represent the cumulative effect of these factors [12]. Hence changes in neutrophil phenotype could be one of the most accurate monitoring opportunities of the immune system currently available. Several reviews focused on neutrophils and post-traumatic complications [13–16]. Since then, however, new data on the post-traumatic immune response and the role of neutrophils in the development of infectious complications have become available. Although several recent reviews discuss the immune response after tissue trauma, the neutrophil kinetics and the pathologic role which neutrophil subtypes might play in these processes, are only slightly touched upon [7, 16]. Furthermore, due to reduced mortality, the clinical scope of interest has shifted from complications shortly after injury to long-term outcomes, such as recurrent infections and functional recovery [17–19]. This review discusses the up-to-date knowledge and hypotheses on the role of neutrophils and their subtypes after trauma in the development of both short-term and long-term clinical outcomes, with a special focus on infectious complications.

## Methods

For this narrative review, a literature search was performed using PubMed, Embase, and Cochrane Library by two authors (LH, RS) in July 2018. English or Dutch articles were included. No restrictions on publication date were applied. When articles were not available in full text, the author was contacted. Cross reference check was used to search for additional articles. Included articles were imported into reference manager Mendeley and duplicates were removed. Articles that were not relevant for this review and articles with low validity were excluded. Data regarding neutrophil (subset) function (e.g., phagocytosis, killing, production of antimicrobials, and NETs), the post-traumatic immune response, and monitoring the immune system were extracted from the included articles.

## Main text

### The innate immune response to severe trauma: losing the balance

**Fig. 1:**
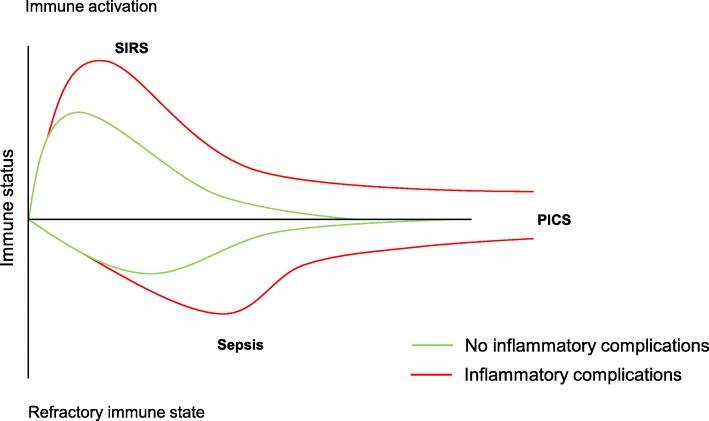
Concept of inflammatory response after trauma. Trauma leads to a rapid immune activation, during which the most competent neutrophils are mobilized into tissues, leaving supposedly less competent neutrophils behind in the circulation. The green lines represent the uncomplicated course after trauma. The immune response can become dysregulated by overwhelming immune activation (upper red line), a refractory immune state (lower red line) or low-grade inflammation and immune impairment later on (both red lines). The vertical axis indicates the immune status. The horizontal axis represents time after trauma. *SIRS* systemic inflammatory response syndrome, *PICS* persistent inflammation, immunosuppression, and catabolism syndrome

The immune response after major trauma is characterized by initial immune activation, followed by a refractory immune state (Fig. 1) [14, 20]. It is thought that the impact of injury and the correlating degree of tissue damage (often denominated by the injury severity score (ISS) [21]) determine the amplitude of these components [22, 23]. Both components seem to be necessary after trauma: the immune activation to resolve tissue damage and combat invading pathogens and the refractory immune state to resolve persistent overwhelming inflammation. However, these components can become dysregulated. Severe injury, especially when accompanied by additional insults such as hemorrhagic shock, mechanical ventilation, and major operative procedures, can cause extensive tissue damage leading to overwhelming immune activation [13, 24]. This overwhelming immune activation (e.g., the systemic inflammatory response syndrome (SIRS)), if severe, can lead to barrier dysfunction and organ dysfunction [14, 25]. It is thought that migration of activated neutrophils into organs is one of the key components in the development of early organ dysfunction (e.g., multiple organ dysfunction syndrome (MODS) and acute respiratory distress syndrome (ARDS)) [].

The following inflammatory response, initially described as anti-inflammatory [28], is characterized by a period in which patients are more susceptible to nosocomial infections, viral reactivations, and sepsis. During this period, patients can develop a septic shock and late, infectious related MODS [14]. Hence, the occurrence of MODS was originally described to have a bimodal distribution caused by either an excessive early pro-inflammatory response or an excessive late anti-inflammatory response [28].

Later it was recognized, however, based on cytokine profiles, leukocyte function, and leukocyte gene expression, that the anti-inflammatory response largely coincided with the pro-inflammatory response after trauma [20, 29–32]. Inflammatory complications were found to be associated with extravasation of activated neutrophils [26, 27, 30] and infections with the presence of less responsive neutrophils in the circulation [8, 33]. For example, neutrophils normally express C5aR abundantly to migrate toward the potent chemoattractant C5a generated in damaged or infectious tissues [34]. However after trauma, neutrophils with a loss of expression of C5aR (CD88) have been found in the circulation of trauma patients [35], especially in those patients who later developed infectious complications or MODS [36, 37]. An explanation for these findings could be that activated neutrophils migrate into damaged tissues leaving the less competent or dysfunctional neutrophils behind. This implies that the susceptibility to infections is not caused by an active anti-inflammatory process, but rather by an immune refractory period caused by loss of more competent neutrophils from the bloodstream. Hence, instead of a “pro-inflammatory response” and an “anti-inflammatory response” as causes of immune-mediated complications after trauma, it might be more accurate to refer to excessive “immune activation” and “refractory immune state,” respectively.

Over the past years, the incidence of severe infections has significantly decreased and in particular late MODS has become rare [38–40]. Also, mortality due to severe infections has dramatically decreased over time [38]. Since more trauma patients survive the first weeks after trauma, especially when treated by trauma experts, nowadays more attention is drawn to the clinical course after this first critical period [4, 6, 41, 42]. A small portion of patients that survive this period now progress to a state of persistent immunosuppression, characterized by a long intensive care unit (ICU) stay and recurrent nosocomial infections [6, 43]. This phenomenon is often observed in combination with persistent inflammation and increased protein catabolism and is thus called the persistent inflammation, immunosuppression, and catabolism syndrome (PICS) [42]. Recurrent wound- or fracture-related infections and failure to sufficiently rehabilitate, as seen in these patients, form an increasing problem for both patient and society [17, 42].

### Modulation of neutrophil function as key factor in the post-traumatic immune response

The inflammatory response after trauma is initiated by damage-associated molecular patterns (DAMPs) and microbe-associated molecular patterns (MAMPs) [15]. DAMPs or “alarmins” are endogenous compounds released by cellular injury [44, 45]. MAMPs are non-self-molecules derived from microbial agents that can enter the blood stream after trauma due to contaminated wounds, intestinal injuries, or broken barriers [7, 46]. DAMPs and MAMPs can activate neutrophils through pattern-recognition receptors (PRRs), which induce an amelioration in a range of neutrophil functions [7, 44]. Also, these molecules cause an increase in circulating neutrophils from the marginated pool (neutrophils adhered to vascular endothelium that are not measured in the bloodstream under normal circumstances) as well as from the bone marrow, a process generally referred to as emergency granulopoiesis [47]. Although the mechanisms of emergency granulopoiesis are not well described, it is thought that this process leads to an increased generation of immature and mature myeloid cells in the bone marrow at the expense of lymphopoiesis and erythropoiesis [4, 48, 49].

After trauma, neutrophils help to resolve tissue damage and to form a sufficient defense against bacteria by phagocytosis, release of antimicrobial molecules through degranulation, and the release of neutrophil extracellular traps (NETs) [7, 50, 51]. Antimicrobial molecules include proteases and reactive oxygen species (ROS). These molecules are essential for bactericidal activity, but can also contribute to collateral organ damage of the host when released into tissues [16]. At the same time, disrupted protective barriers [7] in combination with the presence of immunosuppressive neutrophils [52, 53] and less responsive neutrophils [8, 12, 30, 33, 54], enhance the susceptibility to post-traumatic infections. The knowledge that there are less responsive and immunosuppressive neutrophils in the circulation after trauma is relatively new and not much is known about the clinical implications of these neutrophils. Therefore, specific emphasis on these neutrophil subtypes in relation to inflammatory complications is needed.

### Neutrophil heterogeneity and its role in post-traumatic complications

#### Neutrophil subtypes during inflammation

Neutrophils were traditionally considered a homogenous pool of cells, but lately a growing body of evidence shows heterogeneity within this pool, both morphologically and functionally [55–61]. After severe injury, neutrophils in different maturation stages are rapidly released into the bloodstream, leading to an increase in total leukocyte count [27, 52, 62, 63]. A rapid decrease in leukocyte counts afterwards has been associated with excessive migration of activated neutrophils via activated endothelial surfaces into tissues and septic complications later on [26, 62, 64]. Insight into which neutrophils migrate into tissues and cause further damage, as well as insight into which neutrophils remain in the blood stream and by impaired functions cause increased susceptibility to infections, might reveal diagnostic as well as therapeutic options for inflammatory and infectious complications after trauma.

#### Low-density neutrophils

Numerous studies have aimed to identify neutrophil subtypes during inflammatory conditions [55, 58, 59, 65–71]. Neutrophils with immunostimulatory and neutrophils with immunosuppressive characteristics have been described. In this context, low-density neutrophils (LDN) have gained much attention lately [57, 67, 68, 72, 73]. These neutrophils can be distinguished after density gradient centrifugation of blood from patients with systemic inflammation due to, e.g., trauma, infections, autoimmune diseases, and cancer [57, 74]. After density gradient centrifugation of whole blood, LDNs can be isolated from the peripheral blood mononuclear cell (PBMC) fraction, whereas normal density neutrophils (NDNs) can be isolated from the polymorphonuclear (PMN) cell fraction, the fraction where neutrophils are normally found during homeostasis. LDNs are generally described as activated neutrophils and can be divided into (1) immature neutrophils, (2) immunosuppressive LDNs, also known as the “granulocytic myeloid derived suppressor cells” (MDSCs), and (3) pro-inflammatory LDNs [57]. The definition of the pro-inflammatory LDNs relies on their enhanced or primed release of pro-inflammatory cytokines and NETs compared to NDNs [57, 68]. Paradoxically, in the same fraction of these immune-stimulating neutrophils (and thus with similar buoyancy), the immunosuppressive LDNs are found. These immunosuppressive LDNs are so defined by their ability to suppress T cells (both the proliferation as well as their IFNγ production) through CD18-mediated arginase 1 release and/or ROS [57, 67]. The mature LDNs can be distinguished from the immature LDNs by CD10 expression [67]. It was found that the immature LDNs (CD10^−^), in contrast to the mature LDNs (CD10^+^), were able to promote T cell survival, proliferation, and IFNγ production [67]. Thus, the LDNs form a heterogeneous population of neutrophils with increased buoyancy as common factor. The appearance of these cells is related to inflammatory pathologies and, based on some correlation between disease severity and the amount of LDNs [57], clinical interest in these cells is raised. However, the role these cells might play in the immune processes after trauma needs to be further elucidated.

#### VLA-4 positive neutrophils

Another distinct neutrophil subset that was lately identified in mice was a subset of very late antigen-4 (VLA-4 (CD49d/CD29)) positive pro-angiogenic neutrophils [55]. These neutrophils exhibit a higher expression of vascular endothelial growth factor receptor 1 (VEGFR 1) and C-X-C chemokine receptor type 4 (CXCR4) [75] and recruitment of these cells to hypoxic tissues is associated with enhanced vessel growth [76]. VLA-4 positive neutrophils have also been identified in a murine model of infection by Tsuda et al. [58]. In this study, PMN-I (CD49d^high^CD11b^low^) and PMN-II (CD49d^low^CD11b^high^) neutrophils were isolated from methicillin-resistant *Staphylococcus aureus* (MRSA)-resistant hosts with mild SIRS and MRSA-susceptible hosts with severe SIRS, respectively. These findings suggest that the presence of CD49d^high^CD11b^low^ neutrophils decreases the susceptibility to MRSA infections. The PMN-I and PMN-II neutrophils exhibited differences in expression of Toll-like receptors, cytokine patterns, and interaction with macrophages, indicating that these neutrophils were derived from functionally different neutrophil subtypes. Although the clinical significance in humans is yet to be determined, especially in the context of trauma, these findings do underscore the functional relevance of neutrophil heterogeneity.

#### Neutrophil subtypes in relation to trauma

Neutrophil subtypes that have been studied in human after trauma include subtypes based on neutrophil maturation. Under homeostatic conditions, there is only a homogeneous population of mature neutrophils (CD16^bright^/CD62L^bright^) with a segmented nucleus circulating in the peripheral blood. After trauma however, large amounts of immature neutrophils with a banded shaped nucleus (CD16^dim^/CD62L^bright^) enter the circulation almost immediately [52, 77], and after several days also hypersegmented neutrophils (CD16^bright^/CD62L^dim^) can be observed [52]. In addition, after trauma an expansion is observed of immature myeloid cells with immunosuppressive properties, the myeloid-derived suppressor cells (MDSCs) [78, 79]. It should be noted though that the function of these maturation subtypes is mainly studied in a human endotoxin model and unambiguous translation to trauma might not be accurate.

#### Neutrophil subtypes in the human endotoxin model

The human endotoxin model, in which lipopolysaccharide (LPS) is intravenously administered to healthy volunteers, is a well-accepted model to study acute inflammation such as that found during SIRS [80]. It seems that challenge with LPS (a MAMP) and injury (through DAMPS mainly, but also by MAMPs) both cause a similar increase in neutrophil count [81] and appearance of neutrophil subtypes (banded, segmented, and hypersegmented neutrophils) [52] in the circulation. Regarding neutrophil phenotype; however, both similarities [82] as well as differences [81] between the LPS model and the situation found after injury have been reported. Therefore, caution should be taken in extrapolating experimental findings derived from LPS models to the clinical setting. However, data from LPS studies are often less heterogenic and due to a lack of trauma data, the best available data on this subject.

#### The hypersegmented neutrophil as “Trojan horse”

After trauma, as well as after LPS administration, banded and hypersegmented neutrophils can be observed in the circulation [52, 65]. Based on proteome profiling, banded and segmented neutrophils show similar characteristics [65]. However, banded neutrophils have a 2-day shorter maturation time [65]. Thus, neutrophil maturation seems to coincide with an increase in nuclear lobes. Therefore, it is tempting to speculate that hypersegmented neutrophils are even “older” than segmented neutrophils. However, comparison of proteomes suggests that hypersegmented neutrophils are a truly separate subset recruited to the bloodstream during inflammation [65]. Hypersegmented neutrophils do not only differ in phenotype but also in functionality. Endothelial adhesion, important for neutrophil migration into tissues, is decreased in these cells, probably due to a low expression of L-selectin (CD62L) [83]. Also, it has been described that hypersegmented neutrophils are able to suppress T cell proliferation through release of H_2_O_2_ in the immunological synapse created by the integrin Mac-1 [52]. Lastly, hypersegmented neutrophils were found to be less capable of preventing bacterial outgrowth than banded and segmented neutrophils in a LPS model [53]. Banded neutrophils showed the best antimicrobial capacity, in contrast to what was suggested before [84]. The reason for increased bacterial outgrowth in hypersegmented neutrophils was impaired intracellular killing after adequate phagocytosis, most likely due to a deficit in the acidification of the phagolysosome [53]. If this leads to a release of bacteria back in the circulation, this could have detrimental effects for the host. The hypothesis that hypersegmented neutrophils may act as “Trojan Horses” during systemic inflammation complies with our observation that severe infectious complications are mainly seen after 5 days post-trauma [33] when there are also hypersegmented neutrophils in the circulation. Neutrophil lifespan is estimated to be approximately 5 days [85, 86]. It is possible that the massive release of neutrophils into the bloodstream immediately after trauma leads to depletion of well-functioning neutrophils 5 days later. Hypothetically, this could lead to the release of inferior hypersegmented neutrophils or a release of immature not adequately functioning neutrophils (progenitors) into the blood stream. It is tempting to speculate that in this case, intravenous application of normally segmented neutrophils with an uncompromised bactericidal activity may be beneficial for the post-traumatic course.

#### Less competent neutrophils after injury contribute to bloodstream infections

Immediately after severe injury, neutrophil activation is observed, characterized by increased expression of activation epitopes (e.g., CD11b) [8, 31, 87] and decreased expression of l-selectin (CD62L) [8, 31]. This is followed by a decrease in activation markers of circulating neutrophils [8, 88, 89] and the presence of highly activated neutrophils in the lungs [12]. This implies that the most activated neutrophils migrate into tissues and the less activated neutrophils are left behind in the circulation, a phenomenon also seen in eosinophils during asthmatic airway inflammation [90]. For example, in the setting of porcine traumatic hemorrhagic shock, the electrophysiological membrane potential of blood neutrophils becomes unresponsive toward potent anaphylatoxin stimuli in comparison to pre-shock neutrophil function [91]. Another finding that supports this hypothesis is that neutrophils in the peripheral blood show decreased responsiveness toward the bacteria-derived stimulus *N*-formyl-methionyl-leucyl-phenylalanine (fMLF) after severe injury [8, 12, 30, 54, 88, 92]. The more severe the injury, the less neutrophils responded to fMLF in the expression of active FcγRII and CD11b [54]. The decrease in neutrophil responsiveness was found to have a strong association with the development of septic shock several days later [33]. These findings support the hypothesis that partly refractory, possibly less competent neutrophils are left behind in the circulation after severe injury and that this enhances the susceptibility to bloodstream infections.

Recently, it was recognized that competitive phagocytosis exists among neutrophils [61]. This refers to the finding that all neutrophils are able to phagocytose bacteria, but some are more potent than others. It seems plausible that the more potent neutrophils represent the neutrophils that enter the tissues after activation, while the less potent neutrophils stay in the bloodstream. It was shown that in the absence of these more potent neutrophils, the less potent neutrophils start phagocytosing bacteria [61]. It is unknown if these neutrophils are able to adequately compensate for the loss of the more potent neutrophils. It is tempting to speculate that these less potent neutrophils are also less competent in bacterial clearance and thus might contribute to the enhanced susceptibility to bloodstream infections after trauma. Further research is needed to investigate which neutrophils exactly remain in the bloodstream and how this relates to post-traumatic infections.

#### Immunosuppressive myeloid cells contribute to long-term inflammatory complications

Lately, a growing body of evidence suggests a role for MDSCs in the pathogenesis of post-traumatic inflammatory complications. MDSCs are a heterogeneous pool of immature myeloid cells [93] that circulate in low numbers in the peripheral blood of healthy individuals, but increase during cancer and (post-traumatic) inflammation [4, 79]. MDSCs seem to have the potential to mature, but are inhibited to do so in the environment of chronic inflammation [78]. These cells were found to promote immunosuppression through the inhibition of T cell proliferation and the production of anti-inflammatory cytokines, suggesting a contribution of the MDSCs to the restriction of the post-traumatic response [69, 94, 95]. On the other hand, MDSCs are also known to be involved in persistent inflammation through the production of ROS, nitric oxide, and myeloperoxidase [69, 94]. During sepsis, the role of MDSCs remains elusive and both protective and harmful effects of these cells have been reported [4, 78]. However, regarding long-term post-traumatic complications, persistent expansion of granulocytic MDSCs seems to have harmful effects mainly. In this stage, these cells are associated with increased mortality, prolonged ICU stay, recurrent nosocomial infections, and poor discharge status [6, 94, 96]. The combination of immunosuppression, persistent low-grade inflammation, recurrent nosocomial infections, prolonged ICU stay, and other adverse long-term outcomes is consistent with the PICS phenotype. Thus, these studies support the concept of a pathophysiologic role of persistent expansion of the granulocytic MDSC pool in the development of PICS.

**Fig. 2:**
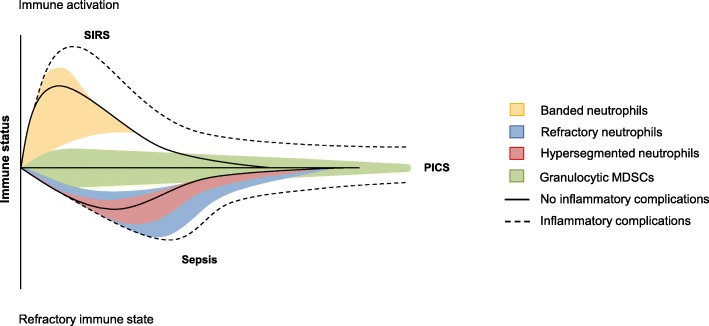
Schematic representation of theories regarding neutrophil subtypes and post-traumatic inflammatory complications. Under homeostatic conditions, there is a homogeneous population of mature neutrophils circulating in the peripheral blood. After trauma, large amounts of immature banded neutrophils enter the circulation. Injury leads to activation of neutrophils and the most activated neutrophils migrate into tissues, leaving less responsive and possibly less competent neutrophils behind in the circulation (the refractory neutrophils). Hypersegmented neutrophils are released into the blood stream after several days post-injury. These cells are known for their decreased bacterial killing after adequate phagocytosis. Therefore, these cells could function as Trojan horses contributing to blood stream infections, especially when present in combination with refractory neutrophils. Also, granulocytic MDSCs with immunosuppressive and immunostimulatory properties are observed after trauma. Persistent expansion of this granulocytic MDSC pool is associated with recurrent infection, prolonged ICU stay, and increased mortality, consistent with the PICS phenotype. *MDSC* myeloid derived suppressor cells, *ICU* intensive care unit, *PICS* persistent inflammation, immunosuppression, and catabolism syndrome

Altogether, these studies support the hypothesis that specific neutrophils subtypes are involved in the pathogenesis of post-traumatic inflammatory complications (Fig. 2). Accumulation of activated neutrophils in organs can contribute to organ dysfunction. At the same time, refractory and possibly less competent neutrophils in the circulation pose a threat in case of invading pathogens, subsequently causing bloodstream infections. Moreover, circulating neutrophils that can release pathogens in the circulation (hypersegmented neutrophils functioning as “Trojan horses”) could increase this risk of infections even further. Long-term inflammatory complications, such as seen in patients with PICS, seem to have a relation with persistent expansion of immature granulocytic cells, the granulocytic MDSCs. These findings underscore the importance of understanding the function and characteristics of various neutrophil subtypes. Although progression has been made in the last decade, much remains to be elucidated.

### Monitoring the immune system after trauma

Understanding the immune system would enable early recognition of patients at risk for inflammatory complications after trauma, which is important since trauma patients can deteriorate quickly. Immune monitoring of trauma patients would provide integrated information about the severity of tissue damage, the reaction of the immune system on this tissue damage, the extent of systemic inflammation, and the development of infectious complications. This information could guide clinicians in decisions regarding damage control surgery and resuscitation, the administration of (preventive) antibiotics and timing of definitive surgery. Even more, this would enable personalized treatment, which is vital since therapeutic measures that modulate the immune response can potentially avert inflammatory complications, but should not be used liberally because the immune response is tightly regulated and inappropriate use of such treatments could lead to further deterioration. It is tempting to speculate that clinicians could use neutrophil immune monitoring to select those patients who would benefit from neutrophil transfusions, transfusions of specific neutrophil subsets, or intracellular antibiotics functioning inside neutrophils that are unable to adequately kill bacteria and as such function as “Trojan horse.”

Nowadays, C-reactive protein (CRP) and leukocyte count are mainly used to monitor the development of infections. However, differences in these markers are usually observed after the onset of infections [27, 97, 98] and to enable early interventions, there is a need for an earlier prognostic marker. Recently, increased neutrophil cell size has been proposed as an early marker, preceding organ dysfunction by two days [27]. The increase in neutrophil size preceded the increase in CRP and neutrophil count [27] and was independently associated with post-traumatic mortality [99]. However, a 2-day interval between change in marker and clinical manifestations is still limited and only a moderate correlation was found between increased neutrophil size at admission and septic complications later on (unpublished results). Several studies attempted to find earlier markers with predictive value for post-traumatic septic complications. Non-neutrophil-related markers that seemed useful included monocyte human leukocyte antigen-DR (mHLA-DR) and procalcitonin (PCT) [97, 100–103]. PCT showed the best results reaching specificity and sensitivity levels around 70% when analyzed 1 day after trauma, and slightly more accuracy when analyzed over time [100]. Hence, PCT could be of value in predicting septic complications, although prognostic accuracy is still limited when used as single indicator [100]. Another disadvantage is that PCT is best analyzed in multiple longitudinal measurements, limiting the window of opportunity between measurements and onset of septic complications. A marker with predictive value found in a univariate analysis as single measurement immediately after injury was fMLF-induced active FcγRII on neutrophils [8, 33]. Expression of neutrophil fMLF-induced FcγRII showed high sensitivity (90%), with rather low specificity (20%) in a heterogeneous trauma population [33]. Although this marker seemed to have negative predictive value immediately after trauma, multivariate analysis for correction of possible confounders is needed to confirm these results. Another prognostic marker found in a cohort of critical care patients was decreased C5aR on neutrophils. A decrease in this receptor within 2 days after ICU admission was a strong predictor for nosocomial infections later on [37]. Although a similar decrease in C5aR has been observed in trauma patients [35], further research should validate the predictive value of this marker in this population. Moreover, there is a need for additional markers to increase prognostic accuracy. Further studies should therefore focus on new biomarkers and combining existing biomarkers to develop an accurate predication model.

### Advances in neutrophil analysis can enable clinical application after trauma

Many neutrophil biomarkers can be analyzed using flow cytometry. During flow cytometry, single cells are measured based on light scattering and fluorescence detection [104]. This enables features like cell counting, measuring cell receptor expression, and measuring intracellular components [104]. In the clinical setting, flow cytometry is frequently used for cell counting and immunophenotyping of lymphocytes in patients with cancer and immune deficiencies [104, 105]. However, flow cytometry is also a useful technique to analyze neutrophils [106]. Characteristics like neutrophil size, neutrophil differentiation (banded, mature, and hypersegmented neutrophils), neutrophil activation markers, responsiveness of these markers to bacterial stimuli, neutrophil viability, and neutrophil phagocytosis can all be measured using flow cytometry [27, 33, 107–110]. Although flow cytometry analysis of neutrophils has proven useful after trauma [33], research on this topic is still limited. Possibly, this is because there are several challenges to encounter:

1. To analyze most neutrophil characteristics other than neutrophil counts, blood needs to be manually processed by experienced laboratory personnel, which roughly takes about 2 h.
2. Neutrophils are sensitive to ex-vivo manipulation, which can cause neutrophil activation and might cause loss of fragile neutrophils during analysis [111].
3. Neutrophil characteristics quickly change over time, thus blood is best drawn as quickly as possible and preferably no later than an hour after trauma [31, 112].

Advances in flow cytometry can presumably overcome these challenges. Recently, flow cytometers with automated sample preparation have become available. These flow cytometers are able to lyse red blood cells, stain leukocytes, count cell types, and analyze marker expression or intracellular staining [108, 113]. Afterwards, analysis results can be automatically transferred into the electronic patient registry. The main advantages of such a closed system are that it does not require any expertise in laboratory techniques such as flow cytometry, that results are very reproducible [113, 114] and that the analysis is quick (15 min instead of ± 2 h). In a clinical setting, laboratories recently started using these automated flow cytometers for lymphocyte analysis, in particular for determining CD4 T cell counts in patients positive for human immunodeficiency virus (HIV) [113, 114]. For neutrophil analysis however, these flow cytometers have never been used before. It is likely that the combination of features these flow cytometer offer will minimize the previously described challenges in neutrophil analysis and will enable neutrophil research on a larger scale, as well as clinical application of neutrophil flow cytometry. With such flow cytometers, it is possible to analyze the aforementioned biomarkers (e.g., CD16, CD62L, C5aR, CD11b, FcγRII, neutrophil size) precisely and within 15 min after blood drawing by any health care worker. Furthermore, the design and features of these machines make it possible to measure samples directly after blood drawing, enabling point-of-care decision making after trauma.

## Conclusion

Trauma leads to a complex inflammatory cascade in which many mediators are involved that induce immune activation as well as a refractory immune state. Both components seem necessary for recovery after trauma. However, overwhelming immune activation or an excessive refractory immune state after trauma can lead to immune-mediated complications. Since neutrophils respond to multiple soluble factors and thus reflect the cumulative effect, neutrophils could be the most accurate read-out of the immune system currently available. Adequately monitoring the post-traumatic immune response would enable early recognition of both short-term and long-term inflammatory complications. Neutrophil-related biomarkers have shown promising results and analysis of these markers is becoming more accessible and applicable. Future research should therefore focus on combining biomarkers to develop an accurate prediction model for post-traumatic inflammatory complications as a first step to improve personalized and point-of-care decision making after trauma.

## Abbreviations

ARDS: Acute respiratory distress syndrome
CRP: C-reactive protein
CXCR4: C-X-C chemokine receptor type 4
DAMPS: Damage-associated molecular patterns
fMLF: *N*-formyl-methionyl-leucyl-phenylalanine
ICU: Intensive care unit
ISS: Injury Severity Score
LDG: Low-density granulocyte
LDN: Low-density neutrophil
LPS: Lipopolysaccharide
MAMPS: Microbe-associated molecular patterns
MDSC: Myeloid derived suppressor cells
mHLA-DR: Monocyte human leukocyte antigen-DR
MODS: Multiple organ dysfunction syndrome
MRSA: Methicillin-resistant *Staphylococcus aureus*
NDN: Normal density neutrophil
NET: Neutrophil extracellular traps
PCT: Procalcitonin
PICS: Persistent inflammation, immunosuppression and catabolism syndrome
PMN: Polymorphonuclear
PRR: Pattern-recognition receptor
ROS: Reactive oxygen species
SIRS: Systemic inflammatory response syndrome
TAN: Tumor-associated neutrophil
TGF-β: Transforming growth factor beta
VEGF-A: Vascular endothelial growth factor A
VEGFR1: Vascular endothelial growth factor receptor 1
VLA-4: Very late antigen-4 (CD49d/CD29)
